# Multiangle Long-Axis Lateral Illumination Photoacoustic Imaging Using Linear Array Transducer

**DOI:** 10.3390/s20144052

**Published:** 2020-07-21

**Authors:** João H. Uliana, Diego R. T. Sampaio, Guilherme S. P. Fernandes, María S. Brassesco, Marcello H. Nogueira-Barbosa, Antonio A. O. Carneiro, Theo Z. Pavan

**Affiliations:** 1 Department of Physics, FFCLRP, University of São Paulo, Ribeirão Preto 14040-901, SP, Brazil; joaouliana@usp.br (J.H.U.); diegothomaz@gmail.com (D.R.T.S.); guilherme.santos.fernandes@usp.br (G.S.P.F.); adilton@usp.br (A.A.O.C.); 2 Department of Biology, FFCLRP, University of São Paulo, Ribeirão Preto 14040-901, SP, Brazil; solbrassesco@usp.br; 3 Department of Medical Images, Hematology and Clinical Oncology, Ribeirão Preto Medical School, University of São Paulo, Ribeirão Preto 14040-901, SP, Brazil; marcello@fmrp.usp.br

**Keywords:** photoacoustic imaging, illumination scheme, in vivo, mouse, Monte Carlo, linear array

## Abstract

Photoacoustic imaging (PAI) combines optical contrast with ultrasound spatial resolution and can be obtained up to a depth of a few centimeters. Hand-held PAI systems using linear array usually operate in reflection mode using a dark-field illumination scheme, where the optical fiber output is attached to both sides of the elevation plane (short-axis) of the transducer. More recently, bright-field strategies where the optical illumination is coaxial with acoustic detection have been proposed to overcome some limitations of the standard dark-field approach. In this paper, a novel multiangle long-axis lateral illumination is proposed. Monte Carlo simulations were conducted to evaluate light delivery for three different illumination schemes: bright-field, standard dark-field, and long-axis lateral illumination. Long-axis lateral illumination showed remarkable improvement in light delivery for targets with a width smaller than the transducer lateral dimension. A prototype was developed to experimentally demonstrate the feasibility of the proposed approach. In this device, the fiber bundle terminal ends are attached to both sides of the transducer’s long-axis and the illumination angle of each fiber bundle can be independently controlled. The final PA image is obtained by the coherent sum of subframes acquired using different angles. The prototype was experimentally evaluated by taking images from a phantom, a mouse abdomen, forearm, and index finger of a volunteer. The system provided light delivery enhancement taking advantage of the geometry of the target, achieving sufficient signal-to-noise ratio at clinically relevant depths.

## 1. Introduction

Photoacoustic imaging (PAI) is a technique based on the photoacoustic (PA) effect, which consists of pressure waves generation due to the absorption of light [1,2,3,4,5]. Currently, laser-based PAI systems use short-duration laser pulses (i.e., ~10^−9^ s) ensuring thermal and stress confinement. As pulsed-light propagates within the target material, its absorption increases the local temperature, causing a thermal-elastic expansion [6] and generating a pressure wave. Thus, PAI encodes the optical absorption information into pressure waves, therefore combining optical contrast with ultrasound spatial resolution [4].

PAI can provide physiological and anatomical information of tissues by accessing their optical, thermal, and mechanical proprieties [5,7]. Since PA signal magnitude is temperature-dependent, PAI has been used, for example, to map temperature variation within tissues during hyperthermia procedures [8,9,10,11]. Moreover, PA magnitude is proportional to the optical absorption of a chromophore; therefore, multi-wavelength PAI is capable of identifying structures with different optical absorption profiles [12,13]. In this context, a typical application of multispectral PAI is to estimate blood oxygen saturation (sO_2_) from the relative concentrations of oxyhemoglobin (HbO_2_) and deoxyhemoglobin (Hb) [14,15,16,17,18,19]. In addition, exogenous contrast agents, for example, nanoparticles and organic dyes, can be accessed to obtain molecular PAI and for drug delivery studies [20]. PAI is frequently combined with clinical ultrasound arrays. This approach allows simultaneously displaying conventional ultrasound images of different modalities (e.g., B-mode and Doppler) with PAI. Different preclinical and clinical, e.g., breast cancer [21] and joint arthritis [22,23], applications of PAI integrated with ultrasound scanners are under investigation.

**Figure 1:**
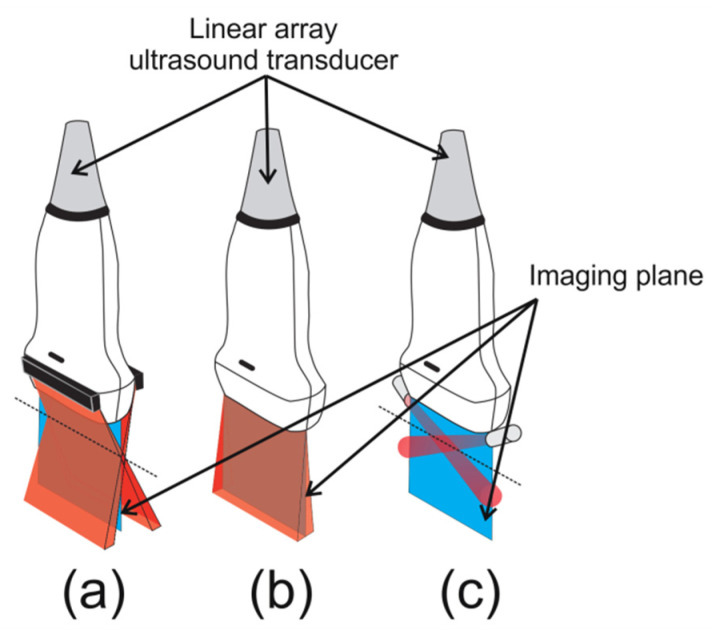
(**a**) Standard dark-field illumination scheme to acquire photoacoustic (PA) image in reflection mode; a rectangular optical fiber terminal illuminates the surface of the target. (**b**) Bright-field illumination where the laser beam and the acoustic field are coaxially aligned. (**c**) Proposed long-axis lateral illumination architecture; the variation of light incidence angle provides wide illumination to the surface of the target.

Hand-held PAI systems usually operate in reflection mode (also known as epi-mode), where illumination and PA wave detection are arranged on the same side [24,25,26,27,28]. For linear array transducers, it is common to illuminate the tissue using a rectangular optical fiber output, which is attached to both sides of the elevation plane (short-axis) of the transducer, see Figure 1a (here this strategy will be referred to as standard dark-field illumination, following the terminology used in [29,30,31,32]). For this dark-field illumination scheme, when the transducer face is in contact with the skin, light is delivered obliquely and relies on light scattering within the target to illuminate the whole field-of-view (FOV) of the transducer. In addition, light absorption outside the imaging plane generates PA waves that can reach the ultrasound transducer. These signals are a source of clutter, which is also an important limiting factor to obtain PA images at deeper regions [33,34,35]. Different studies have investigated, for this standard dark-field illumination scheme, light delivery optimization to enhance PA image contrast and signal-to-noise ratio (SNR) by varying the distance between the fiber output and the transducer and incidence angle between the light beam and the imaging plane [30,33,36,37,38]. In [39], the authors verified, through Monte Carlo simulations, that the optimal illumination configuration depends on the optical properties of the tissues under investigation. They observed that thickness and optical scattering of skin play a major role for this optimization. Another possible strategy to optimize light delivery is to accommodate an optically transparent spacer between the transducer and target’s surface to deliver light directly to the tissue underneath the transducer [25,27,28,32]. Improvements in light delivery could also be achieved by using a concave-shaped light catcher that redirects the light reflected by the skin surface back to the tissue, improving the PA signal magnitude at higher depths [40,41]. The aforementioned studies evaluated laser-based PAI systems. More recently, pulsed light-emitting diodes (LED)-based PAI technique has been proposed as an interesting and cost-efficient option [42,43]. LED-based PAI with linear array usually operates using a similar setup as shown in Figure 1a [42,44]. The study [44] suggested that the high divergence of LED illumination decreases the source direction dependency on PA signal compared to laser.

Since optimal light delivery to the tissue is essential to increase image depth and SNR, custom transducers, new materials, and new strategies have been developed to explore different illumination geometries to improve the quality of the PA image. For example, an ultrasonic transducer fabricated on a glass substrate has an improved transparency allowing the laser beam to propagate through the transducer’s material with low absorption, resulting in overlapped optical excitation and acoustic detection [45]. An ultrasound transducer with a hollow central bore [46] or an optically transparent acoustic transducer [47] could be other options to provide reflection mode illumination. However, these approaches require an extensive redesign of the ultrasound probe and cannot be easily integrated into standard clinical scanners. In epi-mode PAI using standard linear array transducers, optical and acoustic fields can be coaxially arranged (see Figure 1b) by redirecting the laser beam using an optical/acoustic coupler [48] or by using a single or double acoustic reflector to redirect the acoustic waves [29,31,49]. Another strategy for coaxial illumination consists of a custom linear array transducer where the optical fiber outputs and piezoelectric elements are linearly and alternately arranged [50]. These studies [29,31,48,49,50] showed that this illumination strategy improved light delivery when compared with the standard dark-field approach. In the present paper, this strategy will be referred to as bright-field illumination, following the terminology used in [30,31,32,49]. 

An alternative illumination approach, not yet investigated in the literature, would consist of attaching the fiber bundle terminal ends to both sides of the transducer’s long-axis (from now on we will refer to this technique as long-axis lateral illumination), see Figure 1c. In the present paper, we propose a long-axis lateral illumination scheme as a new epi-mode PAI strategy, where the light is delivered within the imaging plane similarly to the coaxial arrangement. In the first part of the paper, Monte Carlo simulations of photon propagation were used to compare light delivery for different illumination strategies; i.e., standard dark-field, long-axis lateral, and bright-field illumination. A transparent spacer positioned between the transducer and the tissue surface was considered for all cases. Tissues with three different geometries were simulated; i.e., targets larger and smaller than the lateral dimension of the imaging plane simulating the human forearm and index finger, respectively and an intermediate situation simulating the cross section of a mouse torso where the abdomen was smaller than the image width and the lower limbs fitted the transducer FOV. The simulations demonstrate that the lateral illumination strategy can provide remarkably improved fluence distribution for targets smaller than the imaging plane.

In the second part of this paper, the development of a simple and easy way to construct a device for long-axis lateral illumination PAI is described. This device employed a nonexpensive commercially available bifurcated optical fiber bundle for light delivery, where no other optical components were required. Since the setup, as shown in Figure 1c, would irradiate only a limited area within FOV, the optical fiber bundle outputs were mounted on movable sockets arranged parallel to the imaging plane to provide multiangle long-axis lateral illumination. The final PA image, covering the full scan area, is then obtained by combining the PA sub-images acquired at different angles. This is a similar strategy as described in [51], where a narrow laser beam scanning approach was proposed for a combined real-time PA-ultrasound imaging system. Then the final PA image was the summation of the sub-images obtained at each laser beam scanning position.

Therefore, this paper presents a novel PAI light delivery where light and sound are coaxially illuminated. Different from other approaches with similar capability [29,31,48,49], the proposed technique does not require an acoustic/optical coupling device. This is an advantage because these coupling modules usually induce important phase distortion to the PA wavefront which can reduce image quality [32]. The light delivery device was prototyped to provide freedom to independently choose the illumination angle, at each side of the transducer, allowing multiangle illumination planning. To show the feasibility of the multiangle long-axis lateral illumination PAI device, images taken from phantom, mouse abdomen, forearm, and index finger of a volunteer were analyzed.

## 2. Materials and Methods

### 2.1. Monte Carlo Simulation of Illumination Schemes

The spatial energy deposition may vary depending on the illumination scheme and target shape. To evaluate the performance of the illumination schemes depicted in Figure 1, light transport was simulated using the MCXLAB Matlab toolbox, which is a 3D voxel-based Monte Carlo model [52], for three different target geometries: (i) a cylindrical target shape simulating a situation similar to what was observed for the human index finger (lateral dimension smaller than transducer’s width); (ii) geometry similar to the human forearm (lateral dimension larger than transducer’s width); (iii) mouse torso as an intermediate case, i.e., part of the target was smaller (mouse abdomen), while the lower limbs of the animal was larger than the transducer’s width.

The standard dark-field illumination scheme shown in Figure 1a, based on the setup described in [53], was composed of two optical fiber terminals (38 mm × 1.25 mm) with the same width as the ultrasound linear array used in the experiments of the present paper. Each terminal was positioned so that the light beam incident angle was 20° and the light beams overlapped at the upper surface of the target. For all three illumination schemes, an optically transparent spacer of 19.5 mm (i.e., water) was positioned between the transducer and the target. For the bright-field illumination scheme shown in Figure 1b, the laser beam was coaxial with acoustic detection. In this case, the illumination dimension hitting the target was 38 mm × 5 mm, which is in accordance with [48]. For the long-axis lateral illumination, Figure 1c, the fiber optic bundle terminals were circular in shape with 5 mm diameter. To illuminate the entire transducer FOV, the same multiangle illumination strategy used for the experiments (see next sections for a detailed description) were adopted in the simulations. We verified that at least 5 laser beam incident angles were necessary to ensure a complete illumination. In this case, all simulation parameters were the same as the experimental setup. A total of (5.0 × 10^6^) photons were used to simulate each situation.

The volume dimension for all simulations was 89 mm × 60 mm × 30 mm with a voxel size of 0.25 mm. The volume consisted of two different materials, the background (water) and an inclusion (target) to simulate the tissue. The optical proprieties of the background were: absorption coefficient $\mu_{a}^{bkg}=3.5640\times 10^{-5}\;{\mathrm{mm}}^{-1}$, scattering coefficient $\mu_{s}^{bkg}=1.0\;{\mathrm{mm}}^{-1}$, $g_{bkg}=1$, and $\eta_{bkg}=1.37$; where g denotes the anisotropic factor and η denotes the refraction index. For the target, the optical scattering and optical absorption coefficients were chosen for a generic tissue, following the equations [54]:

(1) $${\mu^{\prime }}_{s}=a{\left(\lambda /500nm\right)}^{-b},$$

(2) $$\mu_{a}=BO\mu_{a}^{HbO_{2}}+B\left(1-O\right)\mu_{a}^{Hb}+W\mu_{a}^{water}+F_{a}\mu_{a}^{lipid}$$

where ${\mu^{\prime }}_{s}$ denotes the reduced scattering coefficient, $\mu_{a}$ denotes the absorption coefficient, B denotes the average blood volume fraction, O is the oxygen saturation of blood, W is the water content, and $F_{a}$ is the fat content. Selecting a generic tissue composed of 15% of blood at 75% of oxygen saturation, 20% of water, and 10% of fat results in $\mu_{a}^{tissue}=0.062\;{\mathrm{mm}}^{-1}$ at 800 nm. The a and b values were chosen as the mean values estimated for soft tissues ($a=1.89\;{\mathrm{mm}}^{-1}$, b=1.286) [54] resulting in ${\mu \prime }_{s}^{tissue}=1.033\;{\mathrm{mm}}^{-1}$ at 800 nm. The anisotropic factor ($g_{tissue}$) and the refraction index ($\eta_{tissue}$) were 0.95 and 1.37, respectively.

### 2.2. Device for Multiangle Long-Axis Lateral Illumination 

The PA system is composed by an Nd:YAG Laser (Brilliant B, Quantel Laser, Les Ulis, France) and an Optical Parametric Oscillator (MagicPRISM OPO, Opotek, Carlsbad, CA, USA) connected to a trifurcated optical fiber bundle (Oriel Glass Fiber Optic Bundle; numerical aperture 0.56; core diameter: 7.9 mm (common), 5.5 mm (legs); fiber length 36 in; Newport, Irvine, CA, USA). One terminal end of the optical fiber bundle was connected to the sensor of an energy meter (FieldMax II-TOP, Coherent, Santa Clara, CA, USA) providing the measurement of laser fluence in real-time. The other two terminals were used to illuminate the sample. PA and ultrasonic radiofrequency (RF) data were acquired using a commercial ultrasound system (SonixOP, Ultrasonix Medical Corp., Richmond, BC, Canada) connected to a parallel acquisition receiver module (SonixDAQ, Ultrasonix Medical Corp., Richmond, Canada), operating at a sampling frequency of 40 MHz.

**Figure 2:**
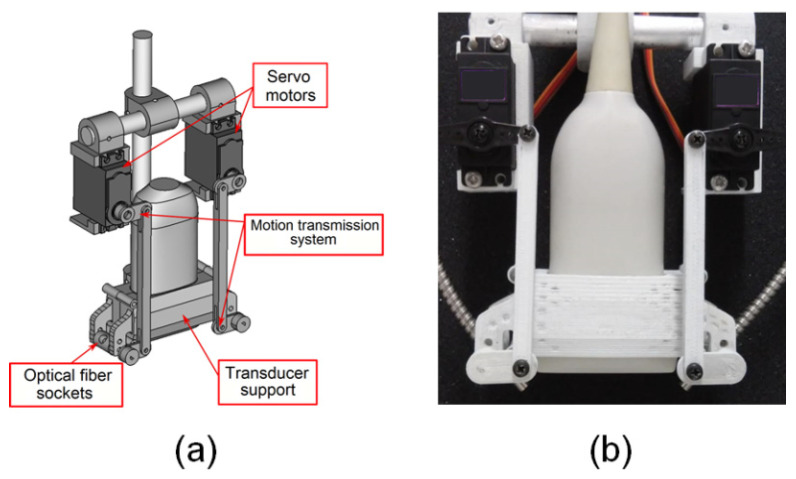
(**a**) Three-dimensional model and (**b**) prototype of the multiangle long-axis lateral illumination device attached to a linear array ultrasound transducer.

**Figure 3:**
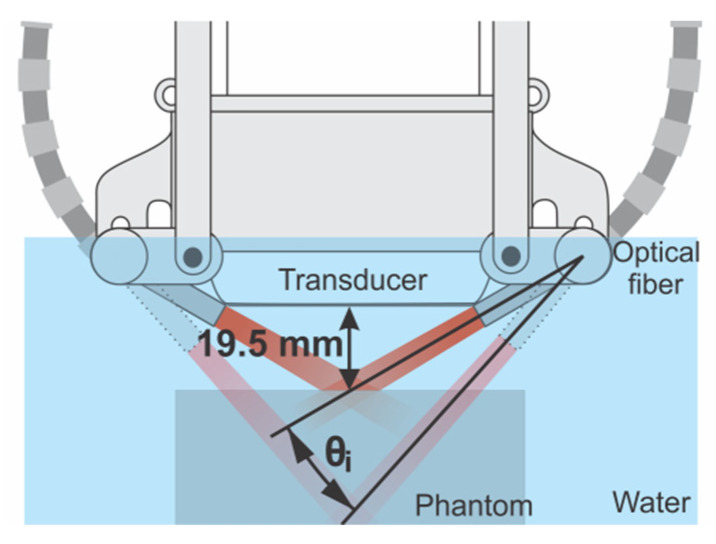
Depiction of the experimental setup used to acquire the multiangle long-axis lateral illumination PA images. The distance of 19.5 mm between the transducer and the phantom surface forces the focal illumination region to be at the phantom surface for $\theta_{i}$ = 0°.

The device for multiangle long-axis lateral illumination was developed using a new architecture that differs from most used configurations presented in previous studies [9,23,24,25,26,33,34,35,36,37,38]. The optical fiber bundle terminal ends were attached to movable sockets placed on the lateral sides of the transducer’s long-axis (see Figure 1c), so that different focal illumination spots within the transducer FOV could be obtained by varying the incident laser beam angles (see Figure 2 and Figure 3). 

The device consists of three main parts: the ultrasound transducer support, the motion transmission system, and two motors. The support and the motion transmission system were designed using the FreeCAD open-source parametric modeling software, see Figure 2a, and printed with Acrylonitrile Butadiene Styrene (ABS) plastic using a 3D printer (ZMorph 2.0 SX, ZMorph, Wroclaw, Poland), as shown in Figure 2b. The angle of the movable sockets is controlled by the servo motors (MG996R, Tower Pro, Shenzhen, China) connected to the motion transmission systems and controlled by an open-source microcontroller (Arduino UNO, Arduino, Turin, Italy).

A linear L14-5/38 ultrasound transducer (Ultrasonix Medical Corp., Richmond, Canada) with 128 piezoelectric elements and a nominal center frequency of 7.2 MHz, was positioned inside the support, which was then attached to the 3D linear stage (HSC-103, Sigmakoki, Tokyo, Japan). A LabVIEW virtual interface (National Instruments Corp., Austin, TX) was developed to control the position of the device as well as the illumination angles. The timing sequence of the synchronous RF data acquisition and multiangle illumination consists in acquiring a pair of PA and B-mode images for each laser pulse (laser repetition rate is 10 Hz). After K laser pulses (K·100 ms) the illumination angle is incremented, the process is then repeated for n illumination angles. A 3D volume is obtained by moving the transducer along the elevation axis with a 3-axes translational stage.

### 2.3. Coherently Summing the PA Subframes

The RF data were acquired intercalating the laser pulse with the pulse-echo transmission for obtaining a prebeamformed PA subframe and then a prebeamformed B-mode frame. The PA sub-image and B-mode image were generated with the delay and sum technique. In PA images, the RF signal $s\left(x_{i},t\right)$ represents the pressure waves generated by the light absorbers and detected by the *i*-th transducer element. The PA wave time of flight from the absorber position to each element of the array is

(3) $$\delta \left(x,x_{i},\;y\right)=\sqrt{y^{2}+{\left(x-x_{i}\right)}^{2}}/c$$

where x and y are the lateral and axial position of the pressure wave source, respectively, while $x_{i}$ denotes the lateral distance of the *i*-th element of the array to the central element. The delay and sum technique for PA subframe reconstruction consists in applying a delay $\delta \left(x,x_{i},y\right)$ to the RF signal $s\left(x_{i},t\right)$ detected by the elements of the transducer and adding coherently [51]

(4) $$S\left(x,y\right)=\sum_{x-\alpha }^{x+\alpha }s\left(x_{i},\delta \left(x,x_{i},\;y\right)\right)$$

where α is the aperture of receive beamforming, i.e., the number of adjacent elements summed.

The coherent sum of reconstructed PA subframes acquired using each illumination angle, without other processing steps, is equivalent to a reconstructed PA image acquired using a wide illumination due to the linear behavior of the delay and sum operation (Huygens–Fresnel principle) [51]. Thus, PA signals reconstructed using the delay and sum technique can be coherently added to gather the contribution of each illumination angle as:

(5) $$S_{C}\left(x,y\right)=\sum_{\theta_{0}}^{\theta_{n}}S_{\theta_{i}}\left(x,y\right),$$

where $S_{c}\left(x,y\right)$ is the reconstructed RF signal coherently summed (PA final image), $S_{\theta_{i}}\left(x,y\right)$ is the reconstructed RF signal acquired at the *i*-th illumination angle (PA subframe), $\theta_{n}$ is the maximum illumination angle.

### 2.4. Phantom Experiments: Evaluation of Multiangle Long-Axis Lateral Illumination PAI

A cubic phantom with a homogeneous distribution of light absorbers (magnetic nanoparticles) was used to evaluate the multiangle illumination and the PA images. The phantom dimensions were 8.0 cm × 8.0 cm × 3.5 cm, and it was manufactured using a mixture of gelatin (Bloom 250, Gelita, Eberbach, Germany) and agar powder (RM026; Himedia Laboratories-LLC, Kennett Square, USA), diluted at dry-weight concentrations of 4% and 2% of water mass, respectively. Iron oxide nanoparticles (Fe_3_O_4_) with dimensions ranging from 20 nm to 30 nm (Nanostructured and Amorphous Materials Inc., Houston, TX, USA) in the concentration of 0.1% of water mass were added to act as light absorbers. Formaldehyde in a weight concentration of 0.5% of gelatin mass was added to increase stiffness and melting temperature. The phantom was manufactured according to the description in [55,56]. 

To avoid any coupling issues, the experiments were performed using targets immersed in water to guarantee that the gap between the ultrasound transducer and the target was completely filled by an optically transparent coupling medium. However, we believe it would be possible to acquire images using a matching layer. For example, this layer could be ultrasound imaging gel (see, for example, [57]) or a gel pad (see, for example, [58]). This is a topic of ongoing research and should appear in future publications.

The phantom was immersed in a water tank with its surface 19.5 mm from the transducer face. For this condition, the focal illumination region was at the phantom surface for $\theta_{i}$ = 0^°^, considered as the smallest possible angle (see Figure 3). Then, for each illumination angle, two PA subframes were acquired and the angle was varied *n* times in steps of Δθ until the *n*-th angle was achieved

(6) $$\theta_{n}=\theta_{min}+n\Delta \theta .$$

The device moved across the elevation axis to obtain a volume (Table 1), resulting in a total of 360 frames. The PA images were acquired at 720 nm with an average fluence of 15 mJ/cm^2^ at the phantom surface. This wavelength was selected for the phantom experiment because one of the energy peaks of the laser is observed at 720 nm; in addition, iron oxide nanoparticles present higher optical absorption at lower wavelengths within the near infrared spectrum region [59]. For the in vivo experiments, 800 nm was selected because it is the isosbestic point of blood [60]. 

The phantom was assumed to have a homogeneous distribution of light absorbers; the optical attenuation coefficient was calculated measuring the fluence of transmitted light through the layers of the phantom with different thickness. The estimated light attenuation of the phantom was $\mu_{phantom}^{att}=\left(0.133\pm 0.011\right)\;{\mathrm{mm}}^{-1}$. Therefore, an analysis of the light delivery was performed by evaluating the PA signal as a function of axial and lateral directions of an averaged PA image taken over the elevation axis. Since the magnetic nanoparticles at low concentration, which is the case of the present experiment, mainly absorb the light energy, optical scattering was considered negligible for this analysis [59].

We defined image depth as the axial distance between the position of a RF signal inside the target and the target surface, therefore not considering the distance between the transducer face and target surface. The average RF signal of the PA subframes at depths and in lateral direction were evaluated using distinct regions of interest (ROI). Based on the number of elements of the transducer (i.e., 128 elements), we defined a central ROI-1 within FOV, which included the five central elements (62–66) and extended from the phantom surface to the maximum depth, with dimensions 1.5 mm × 25.5 mm. Also, peripheral ROIs (ROI-2 and ROI-3) were defined including two sets of five elements positioned at opposite sides of transducer elements: 5–9 (ROI-2) and 119–123 (ROI-3). ROI-2 and ROI-3 had the same dimensions as ROI-1. In addition, the average PA signal magnitude, in the lateral direction from 0 mm to 2 mm of depth, was calculated for all elements (1–128).

Since the phantom had a homogenous distribution of light absorbers, the PA signal amplitude was related to the amount of light delivered. The quantitative analysis of illumination, in the ROI-1 region, as a function of illumination angle was performed using the mean square root of the RF signal amplitude ($A_{RMS}$) calculated in each PA subframe:

(7) $$A_{RMS}\left(\theta \right)=\sqrt{\frac{\sum_{j=x_{a}}^{x_{b}}\sum_{i=y_{a}}^{y_{b}}{\left(S_{\theta }\left(x_{j},y_{i}\right)\right)}^{2}}{\left(y_{b}-y_{a})(x_{b}-x_{a}\right)}}$$

where $x_{a}$, $x_{b}$, $y_{a}$, and $y_{b}$ are the limits of the ROI-1.

The spatial light delivery information, in the central region of the final PA image, was estimated taking the mean axial position of the RF signal ($\bar{y\;}$) as a function of illumination angle in ROI-1

(8) $$\bar{y}\left(\theta \right)=\frac{\sum_{j=x_{a}}^{x_{b}}\sum_{i=y_{a}}^{y_{b}}\left(\left|S_{\theta }\left(x_{j},y_{i}\right)\right|y_{i}\right)}{\sum_{j=x_{a}}^{x_{b}}\sum_{i=y_{a}}^{y_{b}}\left(\left|S_{\theta }\left(x_{j},y_{i}\right)\right|\right)}$$

Thus, $A_{RMS}$ provides information about the mean amount of light delivered per illumination angle in the central region of the transducer while $\bar{y\;}$ provides spatial information about the mean axial position of generated pressure waves.

The final multiangle PA image SNR was calculated taking the envelope-detected image amplitude [61]:

(9) $$\mathrm{SNR}=\sum_{i}\sum_{j}\left[S_{H}\left(x_{i},y_{j}\right)-{\bar{S}}_{nH}\right]/\sigma_{nH}$$

where $S_{H}$ is the Hilbert transform modulus of the RF signal ($S_{H}=\left|H\left\{S_{C}\right\}\right|$), ${\bar{S}}_{nH}$ and $\sigma_{nH}$ are the average and standard deviation of the background noise in $S_{H}$, respectively.

### 2.5. In Vivo Experiments: Human (Finger and Forearm) and Animal (Balb/C Mouse)

**Figure 4:**
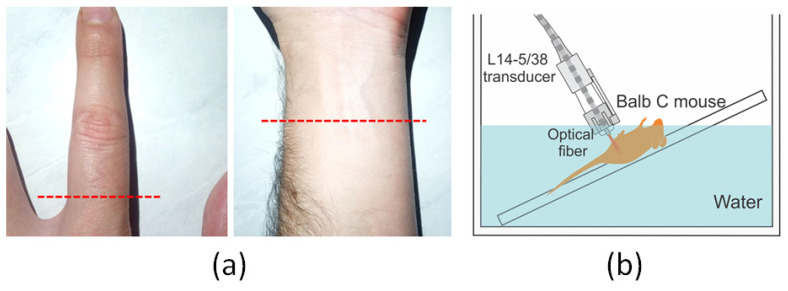
(**a**) Photographs of the index finger and forearm of the volunteer. The dashed lines indicate the position and orientation of the transducer. (**b**) Depiction of the experimental setup used to acquire in vivo PA images of Balb/C mouse.

The index finger of the left hand and left anterior forearm of a human volunteer were photoacoustically imaged using multiangle PAI with illumination parameters according to Table 1. Figure 4a shows photographs of the index finger and forearm where the dashed lines indicate the position and orientation of the transducer. The volunteer immersed his hand and forearm in a water tank; the distance between the transducer face and the skin was chosen so that the laser beams were focused at the skin surface when illumination angle was minimum ($\theta_{i}$ = 0°). The experiments were performed using an average fluence of 9.0 mJ/cm^2^ at 800 nm, obtaining a total of 90 frames.

A male Balb/C mouse, at the age of ten weeks, was anesthetized using vaporized isoflurane (1.0–1.5% isoflurane, Vetflurano, Virbac, São Paulo, Brazil). The animal was positioned in a ramp platform immersed in water with a controlled temperature of 36 °C, as shown in Figure 4b. Using the same wavelength and fluence of the human experiment, PA images of the animal abdomen were acquired.

The experiments involving humans and animals used controlled fluence lower than 20.0 mJ/cm^2^, considering the limit for short-pulse lasers at the skin, which is defined by the American National Standards Institute (ANSI). The animal procedures were approved by the Animal Ethical Committee of Ribeirão Preto Medical School, University of São Paulo (process No. 005/2017-1). The experiments with the volunteer were conducted according to the procedure approved by the Research Ethical Committee of Faculty of Philosophy, Science, and Letters of Ribeirão Preto, University of São Paulo (CAAE: 08860819.4.0000.5407).

## 3. Results and Discussion

### 3.1. Comparison of Illumination Schemes for Different Target Shapes Using Monte Carlo simulation

**Figure 5:**
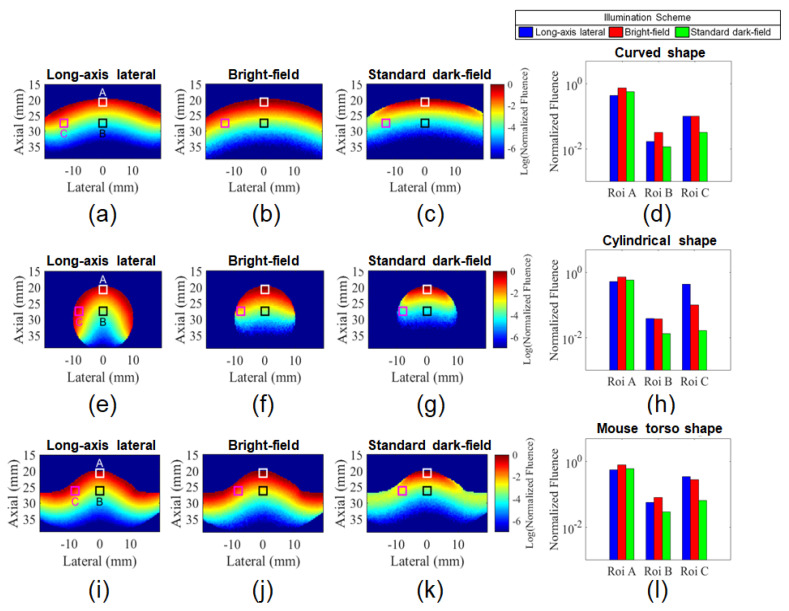
Normalized fluence maps obtained by Monte Carlo simulation for targets larger (**a**–**c**) and smaller (**e**–**g**) than the image width. An intermediate situation representing a mouse torso was also considered (**i**–**k**). All cases were simulated for the bright-field, standard dark-field, and long-axis lateral illumination schemes. Average fluence values were estimated within regions of interest (ROIs) A, B, and C for all cases (**d**,**h**,**l**).

Monte Carlo simulations were conducted to analyze the influence of the target shape and illumination scheme on light delivery. Figure 5 shows the normalized fluence maps obtained for all illumination strategies. Figure 5a–c,e–g show the results for the cases where the target is larger and smaller than the image width, respectively. Figure 5i–k show an intermediate situation representing a mouse torso. In this case, the geometry was obtained by segmenting an experimental B-mode image which will be shown in the next sections. Figure 5a,e,i show the results obtained for multiangle long-axis lateral illumination, while in Figure 5b,f,j, the results for the bright-field illumination coaxial with acoustic detection are shown. Finally, Figure 5c,g,k show the results obtained using the standard dark-field illumination scheme. In these images, ROIs were used to compare the light fluence for different spatial locations (white square is ROI-A; black square is ROI-B and magenta square is ROI-C). Figure 5d,h,l show bar graphs comparing the average fluence estimated within ROIs A, B, and C.

**Figure 6:**
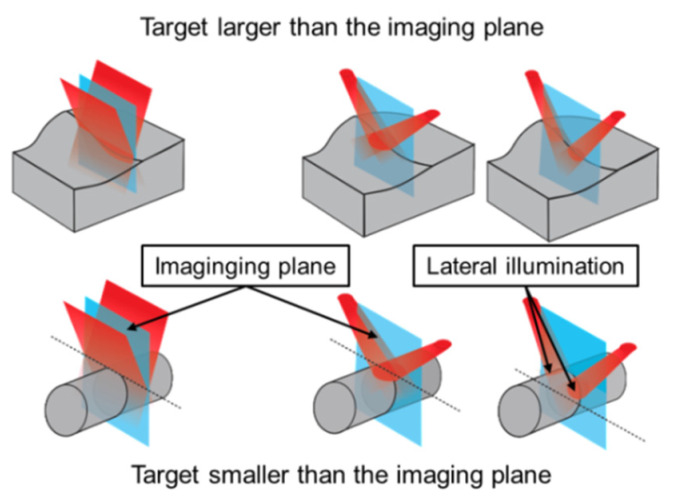
Comparison between the standard dark-field illumination scheme and the multiangle long-axis lateral illumination. For targets larger than the image width with a nonflat surface, the long-axis lateral and the bright-field illumination schemes have the advantage of delivering light within the imaging plane. For targets smaller than the transducer width, the long-axis lateral illumination scheme can deliver light to the sides of the target.

In the central region, all illumination schemes presented similar relative fluence at a shallow depth (ROI-A); yet, to some extent it was consistently higher for the bright-field illumination independent of the target shape. For targets with a nonflat surface, the focus region of the two laser beams used in the standard dark-field arrangement can be partially outside of the material and imaging plane as can be seen on the left side of Figure 6. The light delivered outside of the imaging plane contributes less to the PA image generation and can be a clutter source [62]. In this case, both situations, bright-field and long-axis lateral illumination, have the advantage of delivering light inside the imaging plane even when the target’s surface is not flat. For this reason, the average fluence within ROIs B and C, located at higher depths, was consistently lower for the standard dark-field illumination scenario. It is important to recall that the light beams used for the standard dark-field were overlapping at the tissue surface. Other studies have shown that light delivery can be increased at higher depths, by using a deeper located illumination focus [30,37,38,53]. However, this strategy can dramatically decrease light delivery at shallow depths. For example, the study [48] showed that by positioning the focus at 13.5 mm depth, the simulated fluence estimated for the standard dark-field optical illumination was considerably lower than what was estimated for the bright-field illumination scheme at depths lower than 10 mm. 

For targets smaller than the images’ lateral dimension, the long-axis lateral illumination can deliver light to the sides of the target, increasing the penetration of light inside the material, which is depicted at the right side of Figure 6 and can be observed in the Monte Carlo simulation results. Moreover, the long-axis lateral illumination redirects the light to the target while part of the bright-field and standard dark-field illumination schemes do not contribute to PA signal generation. The simulations show that the relative fluence obtained with multiangle long-axis lateral illumination was dramatically improved for the case of a cylindrical geometry with a diameter smaller than the width of the ultrasound probe, which is a similar situation as the human finger as will be described in the next sections. Figure 5h shows that the average fluence for the long-axis lateral illumination scheme, measured within ROI-C, was four times higher than fluence delivered by the bright-field illumination and one order of magnitude higher compared to the dark-field illumination scheme.

For a target that combines parts smaller and parts larger than image width, as the mouse’s torso, both long-axis lateral and the bright-field illumination schemes provided a relatively uniform light delivery to the entire target surface. On the other hand, the light delivered by the standard dark-field illumination was considerably higher at the top surface. For this situation, the long-axis lateral illumination provided a little increment of fluence within ROI-C compared to the bright-field illumination scheme.

The next two sections aim to evaluate the feasibility of generating PA images using the long-axis lateral illumination scheme. First, the device and the multiangle imaging strategy are evaluated with a phantom experiment; then the possibility of generating the PA images, in vivo, of targets with similar geometries adopted for the simulations are verified.

### 3.2. Analysis of Illumination Angles Contribution to the PA Image of the Phantom

**Figure 7:**
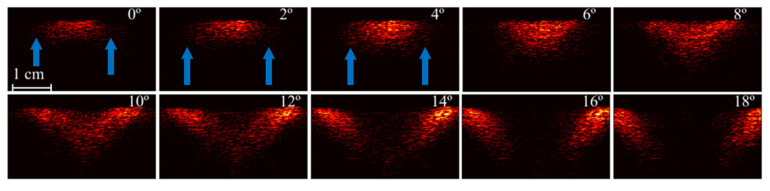
PA subframes of the homogeneous phantom for increasing illumination angles in the range 0°–18°. Each subframe is an average of the phantom’s elevational dimension (i.e., 3.8 cm). Blue arrows indicate the generation of PA signals beyond the laser focal region.

The homogeneous phantom with a flat surface is useful for the characterization of light delivery using different illumination angles. To evaluate the light delivered to the phantom, each PA subframe at $\theta_{i}$ is represented as the average of the 19 PA subframes acquired at different positions of elevation axis (slice) using the same *i*-th illumination angle. The averaged PA subframes in Figure 7 shows the light propagation along depth. The blue arrows indicate the PA signal generated beyond the laser focal region for the illumination angles 0°, 2°, and 4°. This observation can be understood as an advantage of providing illumination from the laterals of the transducer, therefore generating PA signals within FOV for regions not only at the focus. However, the amplitude of the PA signal is a function of the illumination angle, because the laser focus region moves towards higher depth, while the light path increases, reducing the fluence due to light attenuation. 

**Figure 8:**
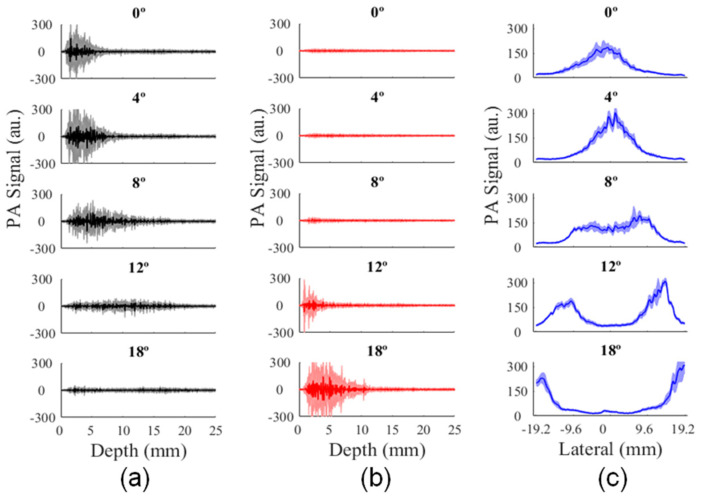
Average PA signal as a function of the illumination angle, along the axial direction at (**a**) central (ROI-1) and (**b**) peripheral ROIs (ROI-2 and ROI-3); (**c**) average PA signal magnitude along lateral direction for depths ranging from 0 mm to 2 mm.

PA signal magnitude increased for depths greater than 10 mm and illumination angles higher than 8°, as it can be seen in Figure 8a. Although illumination along the peripheral areas of the transducer is mostly achieved by just one of the optical fiber outputs, the incident angle of the laser beam in this region decreased relative to the normal surface, delivering light at higher depths, as shown in Figure 8a,b. Moreover, the average PA signal showed the separation of laser beams along the lateral direction; see Figure 8c.

**Figure 9:**
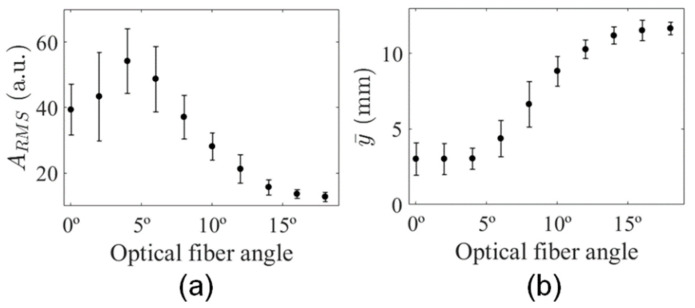
(**a**) Mean square root of PA signal and (**b**) mean depth of PA signal as a function of illumination angle.

The analysis of the $A_{RMS}$ at the central region revealed a proportional decrease in the amount of light delivered for angles higher than 4°, which is probably related to light attenuation within the phantom (Figure 9a). Besides, a peak of maximum $A_{RMS}$ could be observed for $\theta_{i}$ = 4°, showing that the maximum light delivery to the central area occurred when the laser focus region was completely inside the phantom, where the light was less attenuated (shallow depths). These results show the contribution of illumination using $\theta_{i}$ < 4° was less significant for the image of the phantom. In addition, the mean depth of the PA signal increased as a function of the illumination angle (Figure 9b), which could be qualitatively inferred from the plots in Figure 8a. 

**Figure 10:**
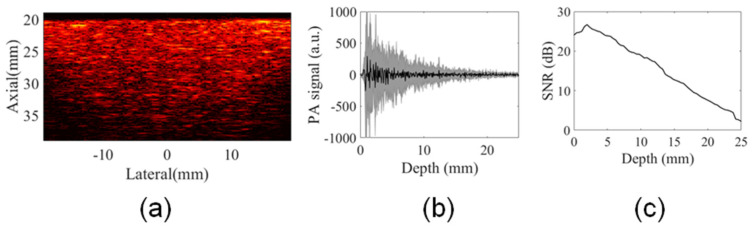
(**a**) Final PA image of a single image slice of the phantom, (**b**) PA signal profile of this PA image shows the contribution of all illumination angles to generate PA signal at depths greater than 10 mm, and (**c**) SNR as a function of image depth.

The final PA image was obtained from the summation of the PA subframes at different illumination angles. For example, a PA image of a single image slice of the phantom is shown in Figure 10a. The average PA signal calculated at depths ranging from 0 mm to 25.5 mm for all elements of this PA image showed the contribution of all illumination angles; see Figure 10b. Figure 10c shows SNR as a function of depth demonstrating the multiangle long-axis illumination PA imaging could be used to perform studies at relevant imaging depths.

### 3.3. In Vivo PA Images

**Figure 11:**
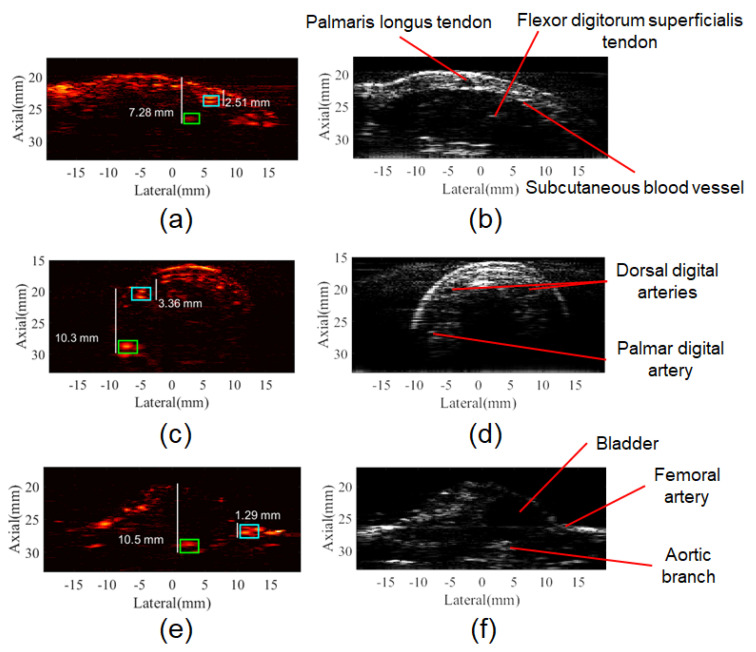
In vivo multiangle long-axis lateral illumination PA and B-mode images. (**a**) PA image and (**b**) B-mode image of the human forearm, anatomical structures such as tendons and subcutaneous blood vessels can be identified; (**c**) PA image and (**d**) B-mode image of the human index finger, the cylindrical shape allows light delivery by the laterals promoting the visualization of the palmar digital artery at depth of 10.3 mm. (**e**) PA image and (**f**) B-mode image of the Balb/Cmouse abdomen, PA signal from an aortic branch at 10.5 mm of depth can be visualized.

Different in vivo experiments were conducted to evaluate the multiangle long-axis lateral illumination PAI. The first in vivo PA images were acquired from a human forearm. In this case, the shape of the surface was larger than the long-axis dimension of the transducer, providing similarities with the flat surface of the phantom and the simulation study. The anatomical structures of the human forearm such as palmaris longus tendon, subcutaneous blood vessels, and epithelial tissue could be identified in the B-mode image as well as in the PA image, as it can be seen in Figure 11a,b. SNR was calculated within two ROIs of 2.4 mm × 1.6 mm in the axial and lateral dimensions, respectively. For the in vivo evaluation, noise ROIs, with the same dimensions, were positioned within 5 mm distance from the structure under analysis. At a tissue depth of 7.3 mm, ROI overlaid the tendon of flexor digitorum superficialis [63] and provided SNR = 14 dB (green rectangle in Figure 11a), while another ROI overlaid a subcutaneous blood vessel and provided SNR = 25 dB at a 2.5 mm tissue depth (cyan rectangle in Figure 11a). Differences in the SNR values between deep and shallow regions were mostly due to light attenuation; the tendon (collagen) also presents an optical absorption coefficient lower than those for melanin or hemoglobin at 800 nm [64], which reduced its SNR on the PA image.

The second acquisition of PA images was obtained from the human index finger, which has a cylindrical-like shape that differs from the forearm or phantom’s surface and has a maximum lateral extension of approximately 20 mm. However, the finger cross-section lateral dimension is smaller than the lateral FOV, allowing illumination from laterals to be more efficient. Consequently, the laser focus becomes deeper while the light path within the tissue is shortened, reducing the light attenuation. SNR was analyzed within ROIs of 2.0 mm × 3.0 mm in the axial and lateral dimensions, respectively. Those ROIs were placed over the location of the dorsal and palmar digital arteries at 3.4 mm (cyan rectangle in Figure 11c) and 10.3 mm tissue depths (green rectangle in Figure 11c), resulting in SNR of 22.5 dB and 22 dB, respectively. 

Lastly, a challenging combination of both aforementioned surface shapes was observed when acquiring the PA image of the cross-section of the mouse abdomen. The abdomen had a lateral dimension smaller than the lateral length of the transducer, while the lower limbs fit FOV. In this case, illumination angles provided light delivery to the sides of the mouse abdomen and hit the surface of lower limbs obliquely. Furthermore, the mouse skin has an average thickness of 0.5 mm and optical absorption lower than human skin [65], increasing light penetration. In the B-mode image of the mouse, the bladder, femoral artery, and a branch of the abdominal aorta can be identified [66]. PA signals from the femoral artery were evaluated at 1.3 mm (cyan rectangle in Figure 11e) and the aortic branch at 10.5 mm of depth (green rectangle in Figure 11e) presenting SNR = 21.5 dB for the femoral artery and SNR = 17.5 dB for the aortic branch (ROIs of 2.0 mm × 2.8 mm).

For three among the most studied PA targets in biomedical applications, the multiangle long-axis lateral illumination was able to provide PA images with high SNR at a depth of 10.5 mm for target shapes with lateral extension smaller than the lateral length of transducer (mouse and index finger). In the human forearm, a tendon at depth of 7 mm generated PA signal with sufficient SNR for good visualization of the structure. However, it should be mentioned that the in vivo PA images were acquired using a measured laser fluence of 9 mJ/cm^2^, which is much lower than the limit for short-pulse laser at the skin. Increasing the laser fluence to values close to the safety limit can improve SNR of PA images at greater depths.

Figure S1 shows the subframes acquired at each illumination angle for the in vivo experiments and plots with the corresponding SNR for ROIs positioned at the selected structures (vessels and tendon). The pronounced variation in the SNR values across the subframes acquired at different angles is evident. Clearly, SNR, at each ROI, observed for the final PA image is similar (only slightly higher) to that subframe with the highest SNR where the illumination area comprised the structure of interest. In the case of combining N PA images at the same illumination angle, it is expected that the SNR will be increased by $\sqrt{N}$, which would be higher than SNR obtained with the multiangle approach for a particular ROI. A more concentrated illumination in the proposed approach, when compared to the techniques illustrated in Figure 1a,b, can also help improve SNR at specific locations of the image. This concept has been also explored in other studies [51,53]. 

Multiangle long-axis lateral illumination could be a useful approach to improve the quality of PA images in preclinical studies with mice, since important anatomic structures are smaller than the FOV. Even for higher frequency linear arrays with a width smaller than the probe used here, murine tumor models still fit in this category [53]. In [53], the authors demonstrated that improving light delivery for this situation can greatly improve PA image quality. Since PA images taken from the human finger joint has shown great potential to evaluate inflammatory arthritis [22,23], it is a possible clinical application where the proposed technique could be beneficial. Future studies will include: (i) evaluating multi-wavelength PA images and fluence correction strategies [57] to monitor blood oxygen saturation and (ii) evaluate the feasibility of using a high divergent source like LED [44] to reduce the number of illumination angles. 

Since the pulse repetition frequency of the laser ($L_{PRF})$ is 10 Hz, the in vivo images of the present study were acquired at a frame rate of 1 frame per second. Each image was composed of two subframes per angle and five different angles were used. The maximum frame rate for this configuration is two frames per second if a single frame was acquired per angle. To acquire the full transducer FOV, a few subframes acquired at different angles are needed; therefore, the frame rate for the proposed technique will be lower than that obtained for the configurations shown in Figure 1a,b. This is a limitation of the proposed approach, especially for the cases where the illumination source operates at low PRF as the laser used in the present study ($L_{PRF}$ = 10 Hz) and monitor fast-changing dynamics is the goal. However, for lasers working at higher repetition rate ($L_{PRF}$ ~ 100 Hz), see, for example, [67]) this limitation can be minimized and the frame rate can be increased. The maximum $L_{PRF}$ supported by the setup depends of the angular velocity of servomotors ($\omega_{s}$), and the step angle (Δθ): $L_{PRF}=\omega_{s}/\Delta \theta$. The servomotors used in the setup can take 0.2 s to rotate the fibers output from 0° to 60°, resulting in a maximum angular velocity of $\omega_{s}=5.2\;rad\cdot s^{-1}$ when operating at 5 V. In this case, the setup could acquire PA subframes using = 75 Hz with Δθ= 4° and provide 15 frames per second when five illumination angles are used. 

The present paper introduced and evaluated the feasibility of the multiangle long-axis lateral illumination to generate PA images, contributing to the development of new illumination strategies in PAI. An advantage of the setup presented here is that it could be additive to other existing illumination schemes. Light delivery by the laterals of the target could be used together with more conventionally used illumination schemes to improve light delivery to targets with lateral dimension smaller than the transducer’s width. The concept of PA images acquired using multiangle illumination can also be applied to setups with similar design as described in [37,38], where the angle of incident light delivered by transducer’s short-axis can be controlled.

## 4. Conclusions

This paper demonstrated the feasibility of using a novel multiangle long-axis lateral illumination PAI. Monte Carlo simulations compared light delivery to tissue for three different illumination schemes: bright-field, standard dark-field, and long-axis lateral illumination. Illumination schemes performance were evaluated for three preclinical and clinically relevant cases for PAI. The shape of the target influenced light delivery for all illumination schemes. Long-axis lateral illumination provided substantial improvement when targets smaller than the lateral width of the transducer were evaluated. The prototype developed to produce multiangle long-axis lateral illumination was evaluated with phantom and in vivo experiments. PA images of good quality were generated from mouse abdomen, forearm, and index finger of a volunteer. Based on the results presented here, a novel PAI system was proposed for preclinical and clinical research. In addition, long-axis lateral illumination could be used together with more conventional illumination schemes to improve light delivery in reflection mode PAI.

## Figures and Tables

**Table 1:** Multiangle illumination and acquisition parameters.

Parameters	Phantom	Finger and Forearm	Balb/C Mouse
Angle step	2°	4°	4°
Elevation step	2 mm	2 mm	2 mm
Image axial	45 mm	35 mm	35 mm
Image lateral	38 mm	38 mm	38 mm
Number of angle steps (n)	9	4	4
Number of elevation steps	19	9	5
Number of frames per angle (K)	2	2	2
Wavelength	720 nm	800 nm	800 nm

## Supplementary Materials

The following are available online at https://www.mdpi.com/1424-8220/20/14/4052/s1, Figure S1: Subframes acquired at each illumination angle used to generate the in vivo PA images shown in Figure 11 of the main article for the (a) forearm, (c) index finger, and (e) mouse abdomen. SNR as a function of illumination angle for each subframe of selected structures located at the (b) forearm, (d) index finger, and (f) mouse abdomen.
