# Supplementary material for: Multiangle Long-Axis Lateral Illumination Photoacoustic Imaging Using Linear Array Transducer
Source: Sensors (Basel). 2020 Jul 21;20(14):4052. doi: 10.3390/s20144052 (PMC7411732; doi:10.3390/s20144052)
Supplement: Supplementary file 1 [file sensors-20-04052-s001.pdf]

sensors

MDPI

Supplementary

Multiangle Long-Axis Lateral Illumination Photoacoustic Imaging Using Linear Array Transducer

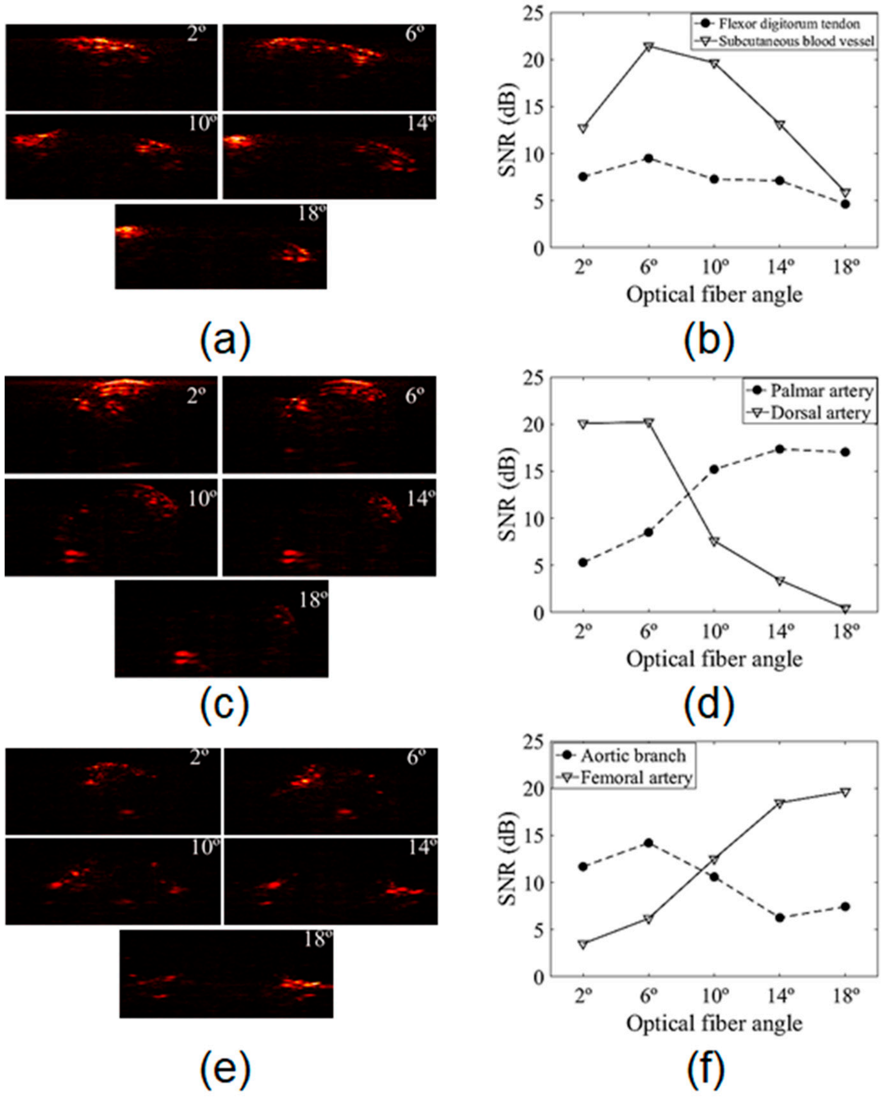

Figure S1. Subframes acquired at each illumination angle used to generate the *in vivo* PA images shown in Fig. 11 of the main article for the (a) forearm, (c) index finger, and (e) mouse abdomen. SNR as a function of illumination angle for each subframe of selected structures located at the (b) forearm, (d) index finger, and (f) mouse abdomen.
